# Contrasting environmental drivers of tree community variation within heath forests in Brunei Darussalam, Borneo

**DOI:** 10.3897/BDJ.12.e127919

**Published:** 2024-12-13

**Authors:** Irsalina Syakirah Mohd Ikbal, Salwana Md Jaafar, Norhayati Ahmad, Rahayu Sukmaria Sukri

**Affiliations:** 1 Environmental and Life Sciences Programme, Faculty of Science, Universiti Brunei Darussalam, Bandar Seri Begawan, Brunei Environmental and Life Sciences Programme, Faculty of Science, Universiti Brunei Darussalam Bandar Seri Begawan Brunei; 2 Institute for Biodiversity and Environmental Research, Universiti Brunei Darussalam, Bandar Seri Begawan, Brunei Institute for Biodiversity and Environmental Research, Universiti Brunei Darussalam Bandar Seri Begawan Brunei

**Keywords:** *Kerangas* forest, forest disturbances, forest management, species diversity, species endemism

## Abstract

Understanding how abiotic factors influence Bornean tropical tree communities and diversity is a key aspect in elucidating the mechanisms of species co-existence and habitat preferences in these biodiverse forests. We focused on investigating forest structure, tree diversity and community composition of lowland Bornean heath forests in Brunei Darussalam, within two 0.96 ha permanent forest plots at Bukit Sawat Forest Reserve and Badas Forest Reserve. All trees with stem diameter ≥ 5 cm were tagged, identified and measured for their stem diameter and basal area. Soil physiochemical properties (pH, gravimetric water content and concentrations of total carbon, nitrogen, phosphorus, potassium, calcium and magnesium in topsoil) and environmental factors (relative humidity, canopy openness, litter depth and topographic variables of elevation, slope and aspect) were quantified as potential drivers of tree community differences. A total of 2,368 trees were recorded, representing 229 tree species in 211 genera and 58 families. Significant between-site differences in forest structure and tree community compositions were detected, despite limited differences in environmental and soil properties. Tree community composition at Bukit Sawat appeared to be influenced by topographic variables, while those at Badas were influenced by canopy openness. Our results showed that small-scale soil and environmental variation appeared to shape the local tree communities at Bukit Sawat and Badas. We recorded numerous Bornean endemic and tree species of high conservation values. We thus highlight the necessity of conducting long-term research on the forest dynamics of Bornean heath forests to effectively manage these high conservation value habitats which are currently experiencing changes driven by disturbances.

## Introduction

Bornean tropical forests contain some of the most biodiverse plant communities worldwide (Kier et al. 2005, Ashton 2014, Sabatini et al. 2022). Tree communities in Bornean forests have been extensively studied (Slik et al. 2003, Slik et al. 2009) and are known to house high numbers of endemic species (Raes et al. 2009, Neo et al. 2020). Various drivers have been attributed to explain species co-existence and the high tree diversity in Bornean forests, with abiotic factors such as topography and soil physicochemical properties often playing key roles in shaping tree communities across local and landscape scales (Cannon and Leighton 2004, Paoli et al. 2006, Sukri et al. 2012, Born et al. 2014, Jucker et al. 2018, Sellan et al. 2019, Limin et al. 2021).

While the factors influencing tree community compositions of widely distributed Mixed Dipterocarp forests existing on nutrient-rich soils have been the focus of most studies (Potts et al. 2002, Russo et al. 2005, Sukri et al. 2012), nutrient-poor Bornean heath forests are less well investigated. Within Southeast Asia, tropical heath forests are the most extensive in Borneo and occupy an estimated 3.3% of its total forest cover (MacKinnon et al. 2013). Bornean heath forests are characterised by highly acidic, nutrient-poor, dry sandy soils (Proctor et al. 1983, Vernimmen et al. 2013, Md. Jaafar et al. 2016, Miyamoto et al. 2016, Maimunah et al. 2019), dominated by smaller, pole-sized trees with the vegetation showing specialised adaptations such as thick, coriaceous and sclerophyllous leaves. Ecological studies have focused on describing their forest structure and plant communities, showing that, while their tree communities are less diverse than the Mixed Dipterocarp forests, their levels of endemism are considerably higher (Brunig 1974, MacKinnon et al. 2013). However, a detailed understanding of the factors influencing these unique heath forest plant communities are still lacking (Ikbal et al. 2023). Bornean heath forest tree species distributions, abundance and diversity are known to be influenced by various factors, such as soil chemistry in Gunung Mulu, Sarawak (Proctor et al. 1983), humus depth in Central Kalimantan, Indonesian Borneo (Miyamoto et al. 2003) and soil nitrogen contents in white sands heath forests in Brunei Darussalam (Din et al. 2015). Most recently, Sellan et al. (2019) found that the distribution, species richness and forest structure of heath forests in Kabili-Sepilok, Sabah were influenced by soil chemistry and topography. Given Brunei's high forest cover, with 54% of its forests still in pristine condition (Bryan et al. 2013), the intact heath forests of Brunei Darussalam thus present an opportunity for an in-depth study of the influence of environmental and soil factors upon its tree communities.

The forest structure, tree diversity and community composition of heath forests in Brunei Darussalam have been increasingly explored (Ikbal et al. 2023). For example, at the Tutong White Sands heath forest, differences in tree species composition were associated with total nitrogen concentrations in the topsoil and percentage canopy openness (Din et al. 2015). At Bukit Sawat and Badas, Davies and Becker (1996) reported that topographic relief and drainage gradient appeared to influence tree species abundance and basal area. Brunei’s heath forests are known to comprise different sub-types, such as Kerangas forests on drier soils, Kerapah forests on waterlogged soils, heath forests on white sands and coastal heath forests, with each sub-type home to different plant communities (Din et al. 2015, Wong et al. 2015, Nafiah et al. 2022), likely reflecting differing habitat preferences that influence these plant communities.

Our study focused on the two heath forest sites previously studied by Davies and Becker (1996) in Brunei, Bukit Sawat and Badas. Although the tree communities at these sites were previously surveyed, little attempt has been made to investigate the potential drivers that shape their tree communities. Given the rapid rates of forest loss throughout Borneo (Gaveau et al. 2014, Estoque et al. 2019), including loss of these rare heath forests (Becek and Odihi 2008), improved understanding of the key drivers affecting tree communities is crucial for their management and conservation (Brunig 2016, Ikbal et al. 2023). Heath forest communities throughout Borneo appear varied in their composition and, thus, a comparison of two such sites will enable localised environmental and soil factors to be examined, providing increased understanding of the role of microhabitat heterogeneity in influencing species diversity and composition. We, therefore, aimed to quantify whether variation in environmental and soil properties influenced forest structure, tree diversity and community composition at Bukit Sawat and Badas. We hypothesised that the tree communities at Bukit Sawat and Badas are distinct, with significant differences in forest structure, tree diversity and community composition and that plot-level soil and environmental heterogeneity are a significant influence upon the tree communities.

## Material and methods

### Study sites

**Figure 1.**
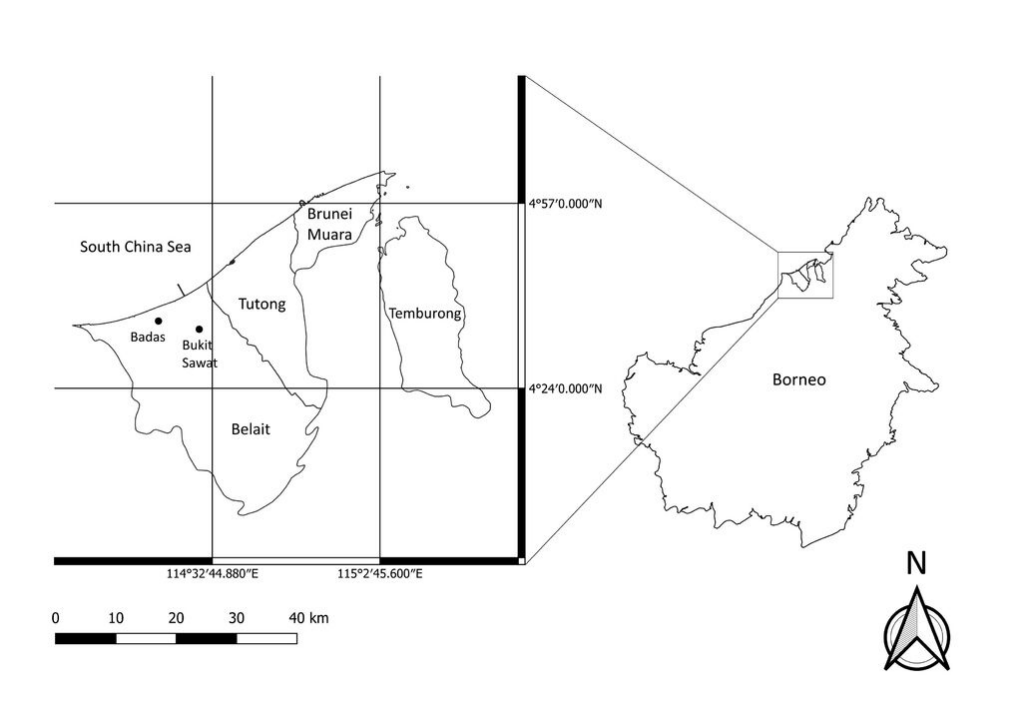
Location of study sites at two 0.96 ha permanent heath forest plot in Bukit Sawat and Badas, Belait District, Brunei Darussalam.

The study was conducted within two 0.96 ha permanent heath forest plots (Fig. 1) in the Belait District, Brunei Darussalam, managed by the Institute for Biodiversity and Environmental Research at Universiti Brunei Darussalam: Bukit Sawat Forest Reserve (4°34'32.27"N, 114°30'24.73"E, elevation 11-23 m) and Badas Forest Reserve (4°56'72.51"N, 114°41'76.45"E, elevation 11-16 m). Both permanent plots were set up in 1992 for long-term monitoring of forest dynamics (see Davies and Becker (1996)). The plot size of 0.96 ha was chosen by Davies and Becker (1996) to take into account the topography of the two sites and we utilised the original grids that were set up for both plots. Plots of 1 ha size are commonly used in similar studies for assessing tree diversity in Bornean heath forests (Proctor et al. 1983, Miyamoto et al. 2003, Sheil et al. 2010) and so, our plot sizes of 0.96 ha, although slightly smaller, are consistent with these studies. These heath forest plots differ in their topography and vegetation composition (Davies and Becker 1996). Bukit Sawat has an undulating terrain with swampy areas with impeded drainage and the tree community is comprised of a mixture of heath and peat swamp forest species. In contrast, Badas occurs on top of a peat swamp dome (Nafiah et al. 2022), showed little topographic relief and the tree community appeared to be comprised of typical heath forest species dominated by *Agathisborneensis* (Araucariaceae).

### Tree census

Each 0.96 ha plot was subdivided into 20 m × 20 m subplots and, within each subplot, all living trees with diameter at breast height (DBH) ≥ 5 cm were tagged, mapped and measured using a diameter tape (Metri, Germany). DBH measurements followed the standard protocols of the Center for Tropical Forest Science (Condit 1998). For each tree, DBH measurement was taken at 1.3 m above the ground. For trees with a bulge at their 1.3 m point, DBH measurements were taken at 5 cm from the lowest point of the bulge. Climbers found on the point of measurement were first removed before determining measuring DBH.

### Species identification

All censused trees were identified to species level in the field with the assistance of botanists from the Brunei National Herbarium (BRUN), Forestry Department. Individual trees that could not be identified to species level were assigned to morphospecies. Voucher specimens were collected and further identified at BRUN for confirmation of taxonomic identification through cross-checking with BRUN specimens. All voucher specimens with confirmed taxonomic identification were deposited in the IBER Herbarium. Selected voucher specimens were also identified by cross-checking against specimens at Royal Botanic Gardens Kew.

### Soil sampling and analysis

Within each 20 m × 20 m subplot, five soil cores were collected i.e. one from every corner and one in the centre from topsoil (0 – 15 cm depth) using a soil auger. The five samples per subplot were combined to form a composite bulk for each subplot, resulting in a total of 24 soil samples collected per site. All soil samples were collected within one week for each study site, during the dry period in August 2022.

Fresh soil samples were analysed for soil pH and soil gravimetric water content (GWC; Allen et al. (1989)). Soil pH was determined following Allen et al. (1989), by mixing 10 g of fresh soil with 20 ml of distilled water (soil: distilled water in the ratio 1:2). The soil solution was stirred for five minutes and left standing to allow sediments to settle before the pH of the soil suspension was measured using a benchtop pH meter (Orion Star A211, Thermo Scientific, USA). Gravimetric water content (GWC) was determined by obtaining the dry mass of 10 g of fresh soil after oven-drying for 24 hours at 105°C. The remaining fresh soil samples were air-dried at room temperature (25ºC) for 3 to 4 weeks. Air-dried soil samples were passed through a 2.0 mm sieve to remove any plant debris and ground into fine powder using a ball mill (Mixer Mill, MM400, Retch, Germany) to obtain fine soil samples. The finely ground soil samples were analysed for total C, N, P, Ca, Mg and K concentrations. Total C and N were measured simultaneously on a Leco CN928 Series combustion analyser (Leco, MI, USA). Total P, Ca, Mg and K were dissolved using the microwave-assisted nitric acid-hydrofluoric acid dissolution method for soil (Siliceous 1996). The digest was analysed spectrometrically for P, Ca, Mg and K on SpectroArcos FHX22 (Spectro Analytical Instruments, Kleve, Germany).

### Measurements of environmental properties

Environmental parameters of topography (elevation, slope and aspect), relative humidity, percentage canopy openness and litter depth were measured. Elevation (m a.s.l.) of each 20 m x 20 m subplot was measured using a Garmin GPSMAP 62s (Garmin Ltd, Taiwan) in five replicates and averaged per subplot, while slope was determined in four replicates along the x-axis and y-axis of each plot using a PM-5/360 PC Suunto Optic Clinometer (Suunto, Finland) and averaged per subplot. Aspect for each plot was determined using KB-14/360 R Suunto Optic Compass (Suunto, Finland) and measured in five replicates and averaged per subplot.

Within each 20 m x 20 m subplot, relative humidity was measured in five replicates using a whirling hygrometer (Elcometer 116A Whirling Hygrometer, Elcometer Ltd., UK) and averaged. Percentage canopy openness was measured in five replicates and averaged per subplot. For litter depth, a 50 cm ruler was used to measure the depth of the litter layer (in cm) following Dent et al. (2006), with five-point samples taken per subplot (four from the corners and one from the centre) and values were averaged per subplot. Within each 20 m x 20 m subplot, all measurements were taken at five random locations. All measurements of environmental parameters and soil collection were conducted within a week for both study sites back in August 2022, at the same time of the day (i.e in the morning) and under comparable weather conditions, to minimise environmental variability.

### Statistical analyses

All data analyses were conducted using R version 3.6.3 (R Core Team 2020). Between-site differences in soil parameters (total C, total N, total P, total K, total Ca, total Mg, soil pH and GWC) and environmental parameters (topography, humidity, litter depth, canopy openness) were determined using separate linear mixed effects (LME) models with the nlme version 3.1-137 package (Pinheiro et al. 2018). Where necessary, these parameters were either arcsine or log10-transformed prior to analysis. The factor “Site” was modelled as the fixed effect, soil and environmental parameters were modelled as the response variables and subplot number was modelled as the random effect. All LME model selections were based on the protocols by Pinheiro and Bates (2009) and Zuur et al. (2011).

Stem diameter measurements were used to determine size class distributions and basal area of trees in different size classes. Basal area was calculated as follows (Avery et al. 2018):

\begin{varwidth}{50in}\begin{equation*}
            Basal Area (B.A.) = π (DBH/2)^2
        \end{equation*}\end{varwidth}

The vegan package version 2.5-7 (Oksanen et al. 2020) in R v. 3.6.3 (R Core Team 2020) was used to calculate species richness, diversity indices (Shannon’s index, Inverse Simpson’s Index and Evenness) per subplot at Bukit Sawat and Badas. Stem abundance, DBH and basal area were also determined at subplot level.

Differences in forest structure (represented by DBH and basal area), stem abundance, species richness and diversity indices between sites were analysed using separate linear mixed effects (LME) models in R version 3.6.3 (R Core Team 2020). The factor “Site” was modelled as the fixed effect, forest structure measurements, stem abundance, species richness and diversity indices were modelled as the response variables and subplot number was modelled as the random effect. All LME model selections were based on the protocols by Pinheiro and Bates (2009) and Zuur et al. (2011). The LME approach was selected as subplots within a plot are spatially autocorrelated and the LME structure can take into account this pseudoreplication by utilising subplot number as a random effect in the model, while a simpler analysis such as an unpaired t-test would have resulted in a violation of non-independent sample requirements for parametric tests.

Non-metric multidimensional scaling (NMDS) ordination using species abundance data and the Bray-Curtis index as the distance measure was used to explore the patterns of tree species communities within the two heath forest sites in relation to soil and environmental variables in R vegan package version 3.6.3 (R Core Team 2020). To determine floristic similarity between forest types, PERMANOVA (Anderson 2001) was conducted using the *adonis* function in the R vegan package version 3.6.3 for species abundance data (Nafiah et al. 2022). The function *pairwise.adonis* was used to conduct pairwise comparisons (Martinez Arbizu 2020).

## Results

### Differences in environmental and soil properties

Our results showed that relative humidity was significantly lower at Badas than Bukit Sawat while percentage canopy openness was significantly higher at Badas than Bukit Sawat (Table 1, Suppl. material 1). For topographic variables, slope was significantly higher at Bukit Sawat than Badas (Table 1, Suppl. material 1). However, litter depth, elevation and aspect did not significantly differ between the heath forests at Bukit Sawat and Badas (P > 0.05). For soil properties, soil GWC, total P and total K concentrations were significantly higher at Bukit Sawat than Badas (Table 1, Suppl. material 1). Mean values for soil pH, total C, total N, total Ca and total Mg concentrations were all higher at Bukit Sawat and Badas, but LME analyses did not detect significant differences in these soil properties between sites for (P > 0.05).

### Differences in forest structure (abundance, tree density, stem diameter, basal area and size class distributions)

A total of 2,368 individual trees of DBH ≥ 5 cm were censused in the two 0.96 ha heath forest plots at Bukit Sawat (n = 1,316 trees) and Badas (n = 1,052 trees). Mean tree density (dF = 1, F = 14.48, P < 0.001) and mean abundance (dF = 1, F = 14.48, P < 0.001) were significantly higher at Bukit Sawat than Badas, while mean basal area (dF = 1, F = 4.14, P < 0.05) was significantly higher at Badas compared to Bukit Sawat (Table 2, Suppl. material 2). No significant difference was detected for mean DBH between the heath forests at Bukit Sawat and Badas (P > 0.05).

**Figure 2.**
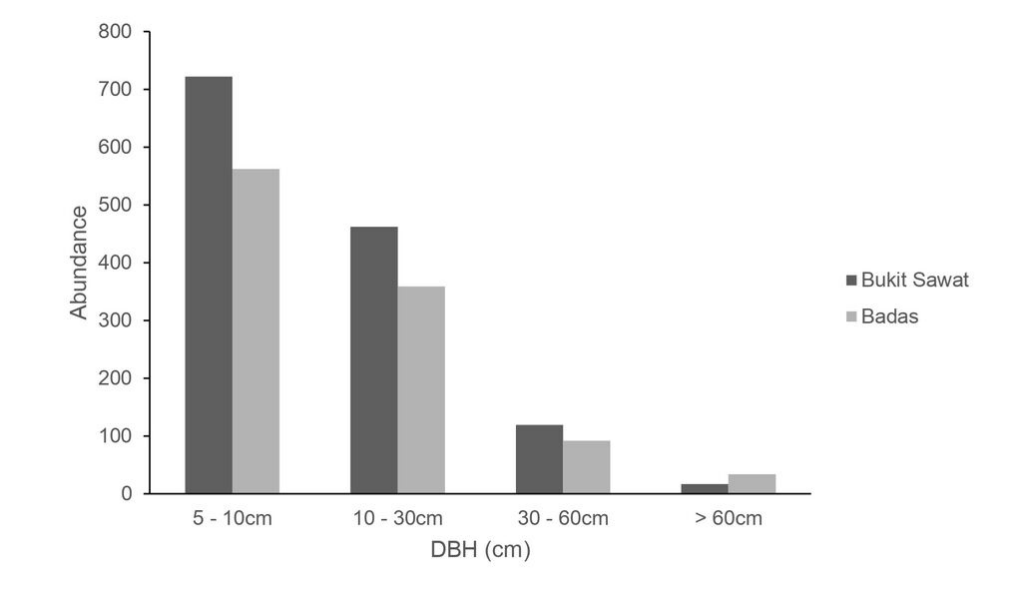
Size class distributions of trees in different DBH (cm) classes in two 0.96 ha plots at the heath forests at Bukit Sawat and Badas.

Bukit Sawat consistently recorded more stems than Badas in all size classes (Fig. 2), except for large trees (DBH > 60 cm) which were twice as abundant at Badas (n = 34 individuals) than at Bukit Sawat (n = 17 individuals). At both sites, small-sized trees (DBH < 10 cm) were most abundant, accounting for over 50% of all stems censused per site.

### Differences in species richness and diversity

A total of 229 species of trees were recorded in this study (Suppl. material 3), representing 58 families and 211 genera. Of this, 190 (83.0%) species were identified to species level, 30 (13.1%) were identified to genus level, five (2.2%) were identified to family level and three species (1.3%) remained unidentified and assigned to morphospecies. A total of 76 species were recorded in both heath forest plots at Bukit Sawat and Badas, 123 species recorded exclusively in Bukit Sawat and 30 species exclusively in Badas (Suppl. material 4). A total of 66 species were recorded as singletons, represented only by a single individual, at Bukit Sawat and 33 species recorded as singletons in Badas.

Total species richness was higher at Bukit Sawat (n = 199 species; 53 families) compared to Badas (n = 106 species; 37 families). At Bukit Sawat, the most species-rich family was Dipterocarpaceae (n = 21 species; 352 individuals), with *Dipterocarpusborneensis* recorded as the most abundant species (n = 80 individuals). At Badas, the most species-rich family was Myrtaceae (n = 15 species; 289 individuals), with *Syzygiumbankense* recorded as the most abundant species (n = 194 individuals). Additionally, at Badas, the tree species *Agathisborneensis* (Araucariacae) was recorded as the second most abundant species (n = 149 individuals). Species richness (dF = 1, F = 92.55, P < 0.001), evenness (dF = 1, F = 57.70, P < 0.001), Shannon’s index (dF = 1, F = 95.55, P < 0.001) and Inverse Simpson’s index (dF = 1, F = 100.10, P < 0.001) were significantly higher at Bukit Sawat than Badas (Table 3, Suppl. material 5).

### Variation in tree communities in relation to habitat variables

**Figure 3.**
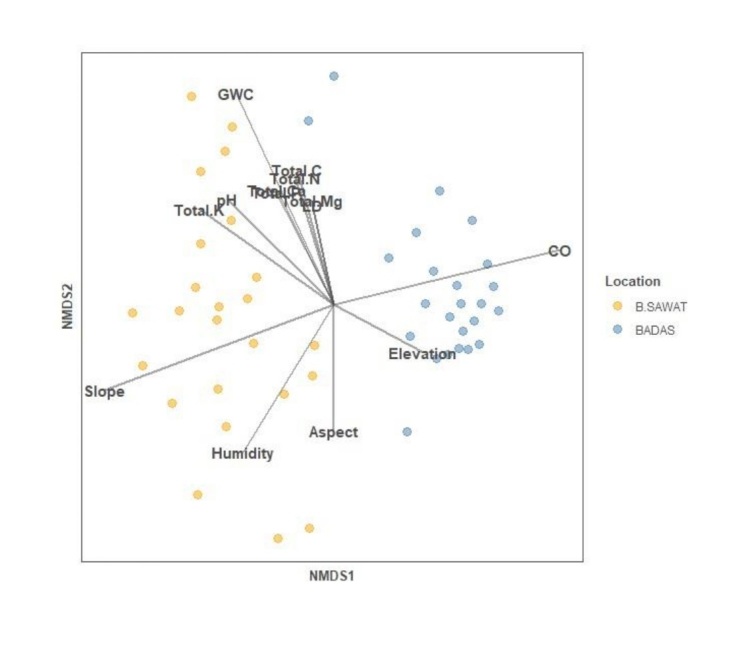
Nonmetric multidimensional scaling (NMDS) of tree communities at Bukit Sawat and Badas 0.96 ha plots using species abundance data in relation to soil and environmental variables. Different colours denote the two heath forest locations: Bukit Sawat (B.SAWAT, n = 24 plots) and Badas (BADAS, n = 24 plots). The direction of the arrow indicates the most rapid change of that variable while the length of the arrow is proportional to the strength of the correlation.

The non-metric multidimensional scaling (NMDS) revealed that tree species composition at the Bukit Sawat and Badas plots were clearly separated out in the ordination space (Fig. 3). The Bukit Sawat plot was significantly influenced by environmental factors (relative humidity and slope) and soil properties (soil pH, soil GWC, soil nutrients, except for total Mg), while the Badas plot was only significantly influenced by canopy openness (Suppl. material 6). The results from PERMANOVA (*F* = 21.84, R^2^ = 0.30, p < 0.001; Suppl. material 7) further showed that tree communities differed significantly between the two heath forest locations.

## Discussion

### Limited differences in environmental and soil properties between Bukit Sawat and Badas

Significant differences in environmental properties between our study sites were only detected for relative humidity, canopy openness and slope. We suggest that the significantly lower relative humidity at Badas is linked to the more open canopy at this site, in comparison to that at Bukit Sawat. The higher canopy cover at Bukit Sawat likely increased sunlight interception, minimising evapotranspiration from the forest canopy and, thus, increasing relative humidity (Russavage et al. 2021). The higher slope at Bukit Sawat reflects its gently undulating topography as opposed to the Badas plot which has a mainly flat topography.

For soil properties, we recorded significantly higher soil GWC and total P and K concentrations at Bukit Sawat than at Badas. We suggest that this is linked to the presence of swampy areas in several subplots at Bukit Sawat (Davies and Becker 1996) which recorded higher GWC and soil nutrients contents. Our recorded GWC values at both heath forest sites were comparable to values recorded from heath forests in Bukit Sawat and Tutong White Sands (Din et al. 2015, Md. Jaafar et al. 2016), while our pH values (3.58 to 3.70) and low soil nutrients contents were consistent with those reported from other Bornean heath forests (Vernimmen et al. 2013, Md. Jaafar et al. 2016, Sellan et al. 2021). Interestingly, our recorded mean values for soil pH, total C, total N, total Ca and total Mg concentrations were all higher at Bukit Sawat and Badas, although these differences were not significant. We note that the peaty topsoil for the swampy subplots at Bukit Sawat contained a thick organic matter layer with a depth of ca. 10 inches (25 cm), while the non-swampy subplots at Bukit Sawat did not have this OM layer. It is possible that within-plot variation resulted in higher mean values for these parameters for Bukit Sawat, but that overall, the soil properties between the two sites did not significantly differ.

### Forest structure variation between Bukit Sawat and Badas

Although no significant between-site differences in mean DBH was detected, both mean tree density and mean stem abundance were significantly lower, while mean basal area was significantly higher, at Badas than Bukit Sawat. We suggest this partly reflects differences in species composition and tree abundance at the two sites. The Bukit Sawat plot was dominated by *Dipterocarpusborneensis* (Dipterocarpaceae) and *Glutabeccarii* (Anacardiaceae), most of which recorded DBH ≥ 20 cm, while the Badas plot was dominated by the tropical conifer, *Agathisborneensis* (Araucariaceae) (Stalin & Franco, 2021), most of which recorded DBH ≥ 30 cm. Similarly, in the Davies and Becker (1996) original study within these same plots, individuals of *G.beccarii* (Anacardiaceae) and *A.borneensis* (Araucariaceae) recorded the largest basal areas in Bukit Sawat and Badas, respectively. Additionally, the Bukit Sawat area has previously experienced low intensity logging (Davies and Becker 1996) which could have resulted in extraction of larger trees with DBH ≥ 30 cm).

Small-sized trees with DBH < 10 cm were the most abundant at both heath forest sites (54.7% of stems at Bukit Sawat and 53.4% of stems at Badas). Other heath forest sites in Brunei (Din et al. 2015, Ikbal et al. 2023) and elsewhere in Borneo (Katagiri et al. 1991, Miyamoto et al. 2007, Sellan et al. 2020) have similarly recorded higher abundance of small-sized trees, which is consistent with the typical characteristic of heath forests having pole-sized trees (Proctor et al. 1983, Wong and Kamariah 1999). We found that large trees (DBH > 60 cm) were twice as abundant at Badas than at Bukit Sawat, consistent with the higher total basal area of *Agathisborneensis* at Badas (Davies and Becker 1996).

### Contrasting tree species richness and diversity between sites

Our findings showed that mean species richness, evenness and diversity indices were significantly higher at Bukit Sawat than Badas. The lower species richness at Badas reflects the dominance of *A.borneensis* (n = 149 trees) and *S.bankense* (n = 194 trees), both of which accounted for 33% of stems recorded at this plot. In contrast, no single species was recorded as exceeding an abundance greater than 100 trees in the Bukit Sawat plot. Consistent with Davies and Becker (1996) who observed lower total species richness at Badas than Bukit Sawat (n = 113 vs. n = 171 species), our study similarly recorded lower total species richness at Badas than Bukit Sawat (n = 106 vs. 199 species). When compared to other heath forest sites within Brunei, our two inland heath forest sites were more species-rich (n = 229 species) than coastal heath forest sites (n = 31 species; Ikbal (2021); n = 61 species) and heath forest at the Tutong White Sands (Din et al. 2015; n = 78 species). Similarly, our sites were more species-rich than heath forest sites in Gunung Mulu National Park, Sarawak (Proctor et al. 1983; n = 113 species), Kabili-Sepilok Forest Reserve in Sabah (Sellan et al. 2019; n = 124 species) and in South Kalimantan (Maimunah et al. 2019; n = 87 species). When compared to non-Bornean heath forests, our sites were similarly more species-rich; for example, studies within the white sands forests in South America have recorded a range of 25 to 147 tree species (Anderson 1981, Klinge and Herrera 1983, Coomes and Grubb 1996, de Oliveira et al. 2014, Adeney et al. 2016), while elsewhere in Southeast Asia, heath forests have recorded a range of 100 to 171 tree species (Davies and Becker 1996, Sellan et al. 2019, Oktavia et al. 2021, Ikbal et al. 2023).

The complete checklist of trees for both plots revealed only 13% of all 229 species censused in this study were found at both sites, indicating distinct tree communities at Bukit Sawat and Badas. Notably, Bukit Sawat recorded several peat swamp species, such as *Dryobalanopsrappa*, *Dactylocladusstenostachys* and *Xylopiacoriifolia*, than at Badas. Consistent with Davies and Becker (1996), the swampy area containing peat soils within our Bukit Sawat plot was able to sustain these peat swamp species. Peat soils have also been recorded in other heath forests of Brunei and Sarawak (Brunig 1974, Wong and Kamariah 1999, Miyamoto et al. 2003). In contrast, the drier soils at Badas were favourable to typical *Kerangas* species, such as *A.borneensis*, *Canariumcaudatum* and *Syzygiumbankense*, all of which were found in high abundance at this site.

### Distinct tree communities and the influence of habitat variables

Although both Bukit Sawat and Badas are heath forest sites, our NMDS ordination and PERMANOVA results have consistently demonstrated that their tree communities were distinct. A crucial finding from our study was the influence of different factors upon the tree communities in these contrasting heath forest sites, as the Bukit Sawat tree community was influenced by a combination of environmental and soil factors. Conversely, the Badas tree community appeared to only be strongly influenced by canopy openness.

The influence of slope at Bukit Sawat is consistent with the initial conclusion of Davies and Becker (1996) that the more heterogeneous topography here appears to increase microhabitat variation when compared to the primarily flat terrain at Badas. Topography is known to strongly influence Bornean tropical tree communities (Aiba et al. 2004, Jucker et al. 2018, Limin et al. 2021) through its associated effects on soil water and nutrient gradients (Baldeck et al. 2013, Weintraub et al. 2015, Chadwick and Asner 2016). The influence of relative humidity, soil GWC and soil nutrients upon the Bukit Sawat tree community likely partly reflects the habitat preference of peat swamp species that were recorded here. Of the 199 species recorded in Bukit Sawat, 60 species (representing 32.7% of censused stems) are known as peat swamp species (Anderson 1980, Coode et al. 1996). Of these 60 peat swamp species, 34 species were recorded exclusively in Bukit Sawat. The extensive areas of peaty soils in the Bukit Sawat plot, estimated to cover 16.7% of the 0.96 ha plot area, were able to house peat swamp species, such as *Copaiferapalustris*, *Combretocarpusrotundatus*, *Dactylocladusstenostachys* and *Drepananthusbiovulatus*, consistent with their habitat range in heath and peat swamp forests (Posa et al. 2011, Nafiah et al. 2022).

Our findings that canopy openness strongly influenced the Badas tree community was somewhat unexpected, as patterns in Bornean tree communities are typically driven by factors such as soil properties and topography. It is possible that canopy openness may be a proxy of disturbance at Badas, as we observed the presence of large forest gaps created from fallen canopy trees, particularly the large-sized *Agathisborneensis* and these forest gaps covered an estimated 25.0% of the 0.96 ha plot area. Forest gaps caused by tree fall are crucial in the forest growth cycle (Brown and Whitmore 1992) and can significantly shape the plant community and influence forest structure as they greatly increase environmental heterogeneity at both spatial and temporal scales (Whitmore 1978, Whitmore 1988, Ashton 1989, Hartshorn 1989). We suggest that the higher occurrence of forest gaps in the Badas plot compared to the Bukit Sawat plot may be indicative of a more dynamic tree community in Badas, although this notion requires further investigation.

### Conservation implications

We highlight that 42 of the 229 tree species (18.3%) censused at our study sites were listed in the IUCN Red List (IUCN 2023). Notably, ten species have high conservation value (HCV): *Hopeamicrantha* (CR), *A.borneensis*, *Cotylelobiumburckii* and *Dryobalanopsrappa* (EN), *Hopeapentanervia*, *Madhucacurtisii* and *Gonystylusaffinis* (VU) and *Dipterocarpusborneensis*, *Brackenridgeapalustris* and *Madhucapallida* (NT) (Table A.3). Additionally, 61 out of 229 species (26.6%) were Bornean endemics, comprising native *Kerangas* or peat swamp species with habitats that are restricted to heath or peat swamp forests, or both.

Although our study sites are in protected areas and the plots were set up as permanent plots for long-term ecological research, signs of anthropogenic disturbances are increasing especially at Bukit Sawat where pioneer species such as *Macaranga* spp. and the invasive *Acaciamangium* have been recorded. Heath forests in Brunei are estimated to account for 50% of Bornean endemism (Ashton 2009). The high endemicity recorded at our study sites signals a crucial need for increased studies on their distributions within these localised habitats and elsewhere in Borneo.

Findings from our study also highlight several possibilities for practical forest management recommendations for these heath forests. At Badas, located within the largest peat dome in Brunei Darussalam, we recommend establishing a comprehensive monitoring programme focused on assessing disturbance impacts, such as tracking changes in canopy openness and its impact on species composition and regeneration and quantifying other disturbance impacts, such as forest fires and subsidence (Addly et al. 2022). Bukit Sawat's proximity to the Labi Road (less than 100 m away) and the resulting ease of access to the forest requires concerted effort to minimise disturbance and avoid further forest fragmentation to protect its microhabitat diversity and plant communities.

Despite the small sampling area of our two plots (total of 3.92 ha), the presence of notable HCV tree species and distinct tree communities emphasise the need to protect all remaining heath forests within Borneo. The tropical heath forests examined in this study, as well as other studies (Proctor et al. 1983, Din et al. 2015, Sellan et al. 2019), all share a remarkable characteristic in that they each harbour a diverse array of endemic and rare species. Conservation strategies should, therefore, incorporate species-specific protection plans and safeguard microhabitats that appear key to driving heath forest tree communities and examine the levels of protection afforded to these increasingly threatened forests. Additionally, controlling the spread of invasive species such as *A.mangium* and mitigating human-induced disturbances are crucial steps in maintaining the ecological integrity of these heath forests.

### Limitations and future research

Despite the valuable insights provided by this study, certain limitations must be acknowledged. Our study focused on two sites within Brunei Darussalam, with limited sampling area and, thus, future work should be expanded to other heath forest locations within Borneo. We focused on quantifying the influence of abiotic factors, but tree communities are also driven by biotic influences (Curran and Webb 2000, Oshima et al. 2014, Suzuki et al. 2016, Aoyagi et al. 2023), which require further study for our sites and elsewhere. Determining the influence of disturbance upon heath forests at our sites and within Borneo is also timely, to better inform and guide the management of these forests. We also recommend long-term monitoring of our plots and other heath forest plots in Borneo, to assess the impact of climate change on forest dynamics. Climate change exacerbate temperature fluctuations, alter rainfall patterns, soil and environmental properties and increase the frequency and severity of droughts or fires, all of which significantly impact forest structure, species composition and population dynamics (Miyamoto et al. 2016, Miyamoto et al. 2021, Davies-Barnard et al. 2023). Given the urgency of understanding climate change impacts and formulating mitigation and adaptation plans to effectively address these impacts, future research should prioritise assessing the resilience and adaptability of heath forests under changing climatic conditions, as well as monitoring potential shifts in species distributions. Long-term ecological monitoring, coupled with detailed climate data, will be essential to formulate adaptive management strategies to preserve and protect these vulnerable ecosystems.

## Conclusions

Our study presented evidence that forest structure, tree diversity and community composition of two lowland heath forests in Bukit Sawat and Badas, Brunei Darussalam are distinct and were influenced by contrasting environmental and soil properties. Microhabitat variation generated by the topographically heterogeneous habitat in Bukit Sawat and the presence of peaty soils, appeared to result in higher species richness and a tree community comprising of heath and peat swamp species. Conversely, the flat topography at Badas likely exerted less influence upon the tree community, which was, instead, influenced by canopy openness likely due to disturbance events linked to forest gaps. We highlight the need for long-term studies into forest dynamics of Bornean heath forests to guide and inform management and protection strategies for these high conservation value habitats.

## Supplementary Material

817085C0-0898-5B31-A63C-038D495034F4 10.3897/BDJ.12.e127919.suppl1 Supplementary material 1 LME results for soil variables
Data type etc. Brief description LME results for soil variables and environmental variables showing the effects of site. File: oo_1020225.docx https://binary.pensoft.net/file/1020225 Irsalina S. Ikbal, Rahayu S. Sukri, Salwana Md. Jaafar, Norhayati Ahmad

04E2D9A2-668C-5117-8B81-3F4C396C2B69 10.3897/BDJ.12.e127919.suppl2 Supplementary material 2 LME results for forest structure variables Data type etc. Brief description LME results for forest structure variables showing the effects of site. File: oo_1020226.docx https://binary.pensoft.net/file/1020226 Irsalina S. Ikbal, Rahayu S. Sukri, Salwana Md. Jaafar, Norhayati Ahmad

FB203BBB-051D-5EA7-A922-DB93264295F2 10.3897/BDJ.12.e127919.suppl3 Supplementary material 3 Checklist of trees recorded in the heath forests Data type etc. Brief description Checklist of trees recorded in the heath forests at Bukit Sawat and Badas, Brunei Darussalam. File: oo_1020227.docx https://binary.pensoft.net/file/1020227 Irsalina S. Ikbal, Rahayu S. Sukri, Salwana Md. Jaafar, Norhayati Ahmad

EFD81DE4-06A3-5299-9B86-FCD4F15CDD55 10.3897/BDJ.12.e127919.suppl4 Supplementary material 4 Venn diagram Data type etc. Brief description Venn diagram showing the species codes for trees with DBH ≥ 5 cm recorded in Bukit Sawat and Badas heath forests arranged alphabetically. File: oo_1020232.docx https://binary.pensoft.net/file/1020232 Irsalina S. Ikbal, Rahayu S. Sukri, Salwana Md. Jaafar, Norhayati Ahmad

90AF0AF1-650B-58B8-A5D5-1AB8C87C9067 10.3897/BDJ.12.e127919.suppl5 Supplementary material 5 LME results for species richness Data type etc. Brief description LME results for species richness and diversity indices showing the effects of site. File: oo_1020228.docx https://binary.pensoft.net/file/1020228 Irsalina S. Ikbal, Rahayu S. Sukri, Salwana Md. Jaafar, Norhayati Ahmad

3EF728B0-EC82-59D4-B852-449B19ADBF38 10.3897/BDJ.12.e127919.suppl6 Supplementary material 6 Fits of four environmental properties and eight soil properties on to NMDS ordinations Data type etc. Brief description Fits of four environmental properties and eight soil properties on to NMDS ordinations of tree species across 48 plots from heath forests at Bukit Sawat and Badas. File: oo_1020229.docx https://binary.pensoft.net/file/1020229 Irsalina S. Ikbal, Rahayu S. Sukri, Salwana Md. Jaafar, Norhayati Ahmad

2FD5AD0E-3263-5DFC-8D5A-4D8691C90ACD 10.3897/BDJ.12.e127919.suppl7 Supplementary material 7 Pairwise PERMANOVA result test results Data type etc. Brief description Pairwise PERMANOVA result test results, based on abundance data, to show pairwise differences in tree community composition between locations: Bukit Sawat and Badas FR. File: oo_1020236.docx https://binary.pensoft.net/file/1020236 Irsalina S. Ikbal, Rahayu S. Sukri, Salwana Md. Jaafar, Norhayati Ahmad

## Figures and Tables

**Table 1.** Differences in mean values of (a) Environmental properties (humidity (%), canopy openness (%), litter depth (cm), elevation (m), slope (°) and aspect (°)) and (b) Soil properties (soil pH, GWC (%), total C, N, P, Ca, Mg, K concentrations (%)) in the heath forests at Bukit Sawat and Badas. Values are mean ± SE, calculated over the total number of subplots within the 0.96 ha plot per site. *, ** and *** indicate a significant difference at p < 0.05, p < 0.01 and p < 0.001 respectively, as analysed using linear mixed effects (LME) model.

**(a) Environmental properties**	**Location**	
	**Bukit Sawat**	**Badas FR**
Humidity (%)	96.0 ± 0.48	91.0 ± 1.40*
Canopy openness (%)	15.3 ± 2.03	33.2 ± 1.21***
Litter depth (cm)	2.18 ± 0.19	1.97 ± 0.15
Elevation (m)	35.7 ± 0.73	37.7 ± 1.12
Slope (°)	8.99 ± 1.09	1.19 ± 0.21***
Aspect (°)	195 ± 21.0	215 ± 15.2
**(b) Soil properties**		
Soil pH	3.70 ± 0.05	3.58 ± 0.04
Soil GWC (%)	20.4 ± 3.35	12.0 ± 1.17*
Total C (%)	3.72 ± 1.97	1.83 ± 0.21
Total N (%)	0.07 ± 0.04	0.04 ± 0.005
Total P (mg g^-1^)	0.03 ± 0.01	0.02 ± 0.001*
Total Ca (mg g^-1^)	0.05 ± 0.01	0.04 ± 0.01
Total Mg (mg g^-1^)	0.05 ± 0.02	0.04 ± 0.004
Total K (mg g^-1^)	0.04 ± 0.01	0.03 ± 0.002*

**Table 2.** Differences in forest structure (mean stem abundance, mean tree density, diameter at breast height; DBH, basal area), total abundance, total tree density and total basal area of trees in the two 0.96 ha plots at the heath forests at Bukit Sawat and Badas. Mean values (± SE) were calculated over the total number of subplots per 0.96 ha plot at each site. * , ** and *** indicate a significant difference at p < 0.05, p < 0.01 and p < 0.001 respectively, as analysed using linear mixed effects (LME) models. Total abundance was calculated as the total number of censused stems within each 0.96 ha plot. Total basal area (m^2^) of a plot was calculated from the sum of basal areas of all censused trees within each 0.96 ha plot.

	Bukit Sawat	Badas FR
Mean stem abundance	54.8 ± 2.22	43.8 ± 1.83***
Mean tree density (per ha)	1369.79 ± 55.57	1095.83 ± 45.78***
Mean DBH (cm)	14.19 ± 0.23	15.34 ± 0.48
Mean basal area (cm^2^)	284.77 ± 14.08	370.84 ± 29.34*
Total abundance	1316	1052
Total tree density	1371	1096
Total basal area (m^2^)	37.13	37.65

**Table 3.** Total species richness, mean species richness and mean diversity indices (Shannon’s index, Inverse Simpson’s index and evenness) in the two 0.96 ha plots at the heath forests at Bukit Sawat and Badas. Mean values (± SE) were calculated over the total number of subplots per 0.96 ha plot at each site. *, ** and *** indicate a significant difference at p < 0.05, p < 0.01 and p < 0.001, respectively, as analysed using the linear mixed effects (LME) model. Total species richness was calculated from the sum of tree species recorded within each 0.96 ha plot.

	Bukit Sawat	Badas FR
Total species richness	199	106
Mean species richness	31.8 ± 0.89	17.8 ± 1.12***
Shannon’s index	3.24 ± 0.03	2.46 ± 0.07***
Evenness	0.94 ± 0.004	0.86 ± 0.01***
Inverse Simpson’s index	20.7 ± 0.96	9.02 ± 0.76***
