# Supplementary material for: Contrasting environmental drivers of tree community variation within heath forests in Brunei Darussalam, Borneo
Source: Biodivers Data J. 2024 Dec 13;12:e127919. doi: 10.3897/BDJ.12.e127919 (PMC11662205; doi:10.3897/BDJ.12.e127919)
Supplement: Supplementary material 1 — LME results for soil variables [file bdj-12-e127919-s001.docx]

Table S1. Results of ANOVA from linear mixed effects model analysis of soil variables (total C, total N, total P, total Ca, total Mg, total K, soil GWC and soil pH) and environmental variables (humidity, litter depth, elevation and canopy openness) showing the effects of site. Significant P-values are highlighted in bold

| Effects | Total C | | | Total N | | | Total P | | |
| --- | --- | --- | --- | --- | --- | --- | --- | --- | --- |
|  | dF | F | p-value | dF | F | p-value | dF | F | p-value |
| Site | 1 | 0.91 | 0.345 | 1 | 0.97 | 0.330 | 1 | 6.77 | **0.012** |

| Effects | Total Ca | | | Total Mg | | | Total K | | |
| --- | --- | --- | --- | --- | --- | --- | --- | --- | --- |
|  | dF | F | p-value | dF | F | p-value | dF | F | p-value |
| Site | 1 | 3.98 | 0.052 | 1 | 0 | 0.998 | 1 | 6.46 | **0.015** |

| Effects | Soil GWC | | | pH | | | Canopy openness | | |
| --- | --- | --- | --- | --- | --- | --- | --- | --- | --- |
|  | dF | F | p-value | dF | F | p-value | dF | F | p-value |
| Site | 1 | 4.86 | **0.033** | 1 | 3.29 | 0.076 | 1 | 55.54 | **< 0.001** |

| Effects | Humidity | | | Litter depth | | | Elevation | | |
| --- | --- | --- | --- | --- | --- | --- | --- | --- | --- |
|  | dF | F | p-value | dF | F | p-value | dF | F | p-value |
| Site | 1 | 10.36 | **0.002** | 1 | 0.60 | 0.443 | 1 | 2.23 | 0.143 |

| Effects | Slope | | | Aspect | | |
| --- | --- | --- | --- | --- | --- | --- |
|  | dF | F | p-value | dF | F | p-value |
| Site | 1 | 93.51 | **< 0.001** | 1 | 0.162 | 0.689 |
