# Supplementary material for: Contrasting environmental drivers of tree community variation within heath forests in Brunei Darussalam, Borneo
Source: Biodivers Data J. 2024 Dec 13;12:e127919. doi: 10.3897/BDJ.12.e127919 (PMC11662205; doi:10.3897/BDJ.12.e127919)
Supplement: Supplementary material 2 — LME results for forest structure variables [file bdj-12-e127919-s002.docx]

Table S2. Results of ANOVA from linear mixed effects model analysis of forest structure variables (mean stem abundance, mean tree density, diameter at breast height; DBH, basal area) showing the effects of site. Significant P-values are highlighted in bold.

| Effects | Mean tree density | | | Mean abundance | | |
| --- | --- | --- | --- | --- | --- | --- |
|  | dF | F | p-value | dF | F | p-value |
| Site | 1 | 14.48 | **< 0.001** | 1 | 14.48 | **< 0.001** |

| Effects | Mean DBH | | | Mean basal area | | |
| --- | --- | --- | --- | --- | --- | --- |
|  | dF | F | p-value | dF | F | p-value |
| Site | 1 | 3.82 | 0.057 | 1 | 4.14 | **0.048** |
