# Supplementary material for: Contrasting environmental drivers of tree community variation within heath forests in Brunei Darussalam, Borneo
Source: Biodivers Data J. 2024 Dec 13;12:e127919. doi: 10.3897/BDJ.12.e127919 (PMC11662205; doi:10.3897/BDJ.12.e127919)
Supplement: Supplementary material 3 — Checklist of trees recorded in the heath forests [file bdj-12-e127919-s003.docx]

Table S3. Checklist of trees recorded in the heath forests at Bukit Sawat and Badas, Brunei Darussalam. Red List Status from IUCN (2023; Least Concern, Near Threatened, Vulnerable, Endangered, Critically Endangered). Endemic status from: A checklist of the flowering plants and gymnosperms of Brunei Darussalam, 1996 and Plants of The World Online (POWO), 2023 ( * Bornean endemic).

| Family | Species | Total tree abundance | | Species Code | IUCN Red list |
| --- | --- | --- | --- | --- | --- |
|  |  | Bukit Sawat | Badas |  |  |
| Achariaceae | *Achariaceae* sp. 1 | 0 | 1 | ACH.SP1 | Not Assessed |
| Anacardiaceae | *Bouea oppositifolia* (Roxb.) Meisn. | 2 | 8 | BOU.OPP | Least Concern |
|  | *Gluta aptera* (King) Ding Hou | 20 | 3 | GLU.APT | Not Assessed |
|  | *Gluta beccarii* (Engl.) Ding Hou | 28 | 0 | GLU.BEC | Not Assessed |
|  | *Mangifera khoonmengiana* Kochummen* | 4 | 3 | MAN.KHO | Not Assessed |
|  | *Parishia maingayi* Hook.f. | 7 | 4 | PAR.MAI | Near Threatened |
|  | *Swintonia schwenkii* (Teijsm. & Binn.) Teijsm. & Binn. | 1 | 0 | SWI.SCH | Not Assessed |
| Anisophyllaceae | *Combretocarpus rotundatus* (Miq.) Danser | 1 | 0 | COM.ROT | Least Concern |
| Annonaceae | *Drepananthus biovulatus* (Boerl.) Survesw. & R.M.K.Saunders* | 4 | 0 | DRE.BIO | Not Assessed |
|  | *Goniothalamus andersonii* J.Sinclair* | 1 | 0 | GON.AND | Not Assessed |
|  | *Maasia glauca* (Hassk.) Mols, Kessler & Rogstad | 4 | 0 | MAA.GLA | Least Concern |
|  | *Maasia hypoleuca* (Hook.f. & Thomson) Mols, Kessler & Rogstad | 2 | 0 | MAA.HYP | Not Assessed |
|  | *Maasia sumatrana* (Miq.) Mols, Kessler & Rogstad | 16 | 0 | MAA.SUM | Least Concern |
|  | *Manoon* sp.1 | 2 | 0 | MAN.SP.1 | Not Assessed |
|  | *Mezzetia havilandii* (Boerl.) Ridl.* | 1 | 0 | MEZ.HAV | Not Assessed |
|  | *Mezzettia umbellata* Becc.* | 16 | 6 | MEZ.UMB | Vulnerable |
|  | *Xylopia caudata* Hook.f. & Thomson | 0 | 1 | XYL.CAU | Not Assessed |
|  | *Xylopia coriifolia* Ridl.* | 3 | 0 | XYL.COR | Not Assessed |
|  | *Xylopia ferruginea* (Hook.f. & Thomson) Baill. | 7 | 0 | XYL.FER | Not Assessed |
|  | *Xylopia malayana* Hook.f. & Thomson | 2 | 0 | XYL.MAL | Not Assessed |
|  | *Xylopia* sp.1 | 1 | 0 | XYL.SP.1 | Not Assessed |
|  | *Xylopia* sp.2 | 2 | 0 | XYL.SP2 | Not Assessed |
|  | sp. 1 | 0 | 2 | ANN.SP.1 | Not Assessed |
| Apocynaceae | *Alstonia iwahigensis* Elmer | 4 | 0 | ALS.IWA | Not Assessed |
| Aquifoliaceae | *Ilex wallichii* Hook.f. | 1 | 0 | ILE.WAL | Least Concern |
| Araliaceae | *Polyscias diversifolia* (Blume) Lowry & G.M.Plunkett | 1 | 8 | POL.DIV | Not Assessed |
| Araucariaceae | *Agathis borneensis* Warb. | 37 | 149 | AGA.BOR | Endangered |
| Burseraceae | *Canarium caudatum* King | 6 | 76 | CAN.CAU | Not Assessed |
|  | *Dacryodes incurvata* (Engl.) H.J.Lam | 9 | 9 | DAC.INC | Least Concern |
|  | *Dacryodes macrocarpa* (King) H.J.Lam | 1 | 0 | DAC.MAC | Not Assessed |
|  | *Dacryodes* sp.1 | 1 | 0 | DAC.SP.1 | Not Assessed |
|  | *Santiria laevigata* Blume | 1 | 0 | SAN.LAE | Least Concern |
|  | *Santiria rubiginosa* Blume | 6 | 0 | SAN.RUB | Not Assessed |
| Calophyllaceae | *Calophyllum ferrugineum* Ridl. | 29 | 4 | CAL.FER | Not Assessed |
|  | *Calophyllum nodosum* Vesque | 4 | 3 | CAL.NOD | Not Assessed |
|  | *Calophyllum sclerophyllum* Vesque | 12 | 0 | CAL.SCL | Not Assessed |
|  | *Calophyllum sundaicum* P.F.Stevens | 1 | 0 | CAL.SUN | Not Assessed |
|  | *Calophyllum tetrapterum* Miq. | 2 | 0 | CAL.TET | Least Concern |
|  | *Calophyllum teysmannii* Miq. | 1 | 0 | CAL.TEY | Not Assessed |
|  | *Calophyllum* sp.1 | 3 | 1 | CAL.SP.1 | Not Assessed |
|  | *Kayea* sp.1 | 4 | 0 | KAY.SP.1 | Not Assessed |
|  | *Mesua* sp.1 | 1 | 0 | MES.SP.1 | Not Assessed |
| Celastraceae | *Kokoona ovatolanceolata* Ridl.* | 8 | 1 | KOK.OVA | Not Assessed |
|  | *Lophopetalum rigidum* Ridl.* | 1 | 0 | LOP.RIG | Not Assessed |
|  | *Lophopetalum* sp.1 | 1 | 1 | LOP.SP1 | Not Assessed |
|  |  |  |  |  |  |
| Centroplacaceae | *Bhesa paniculata* Arn. | 2 | 3 | BHE.PAN | Least Concern |
| Chrysobalanaceae | *Parastemon urophyllus* (Wall. ex A.DC.) A.DC. | 1 | 0 | PAR.SPI | Not Assessed |
| Clusiaceae | *Garcinia bancana* Miq. | 4 | 1 | GAR.BAN | Least Concern |
|  | *Garcinia cf. sarawhensis* Pierre* | 1 | 0 | GAR.SAR | Not Assessed |
|  | *Garcinia cuneifolia* Pierre* | 2 | 0 | GAR.CUN | Not Assessed |
|  | *Garcinia dryobalanoides* Pierre* | 15 | 46 | GAR.DRY | Not Assessed |
|  | *Garcinia gaudichaudii* Planch. & Triana | 2 | 0 | GAR.GAU | Not Assessed |
|  | *Garcinia* sp.1 | 11 | 2 | GAR.SP.1 | Not Assessed |
| Crypteroniaceae | *Dactylocladus stenostachyus* Oliv.* | 2 | 0 | DAC.STE | Not Assessed |
| Ctenolophonaceae | *Ctenolophon parvifolius* Oliv. | 1 | 2 | CTE.PAR | Vulnerable |
| Dilleniaceae | *Dillenia pulchella* (Jack) Gilg | 1 | 0 | DIL.PUL | Not Assessed |
|  | *Dillenia suffruticosa* (Griff. ex Hook.f. & Thomson) Martelli | 2 | 0 | DIL.SUF | Not Assessed |
| Dipterocarpaceae | *Anisoptera marginata* Korth. | 23 | 0 | ANI.MAR | Vulnerable |
|  | *Cotylelobium burckii* (F.Heim) F.Heim* | 21 | 34 | COT.BUR | Endangered |
|  | *Cotylelobium melanoxylon* (Hook.f.) Pierre | 13 | 5 | COT.MEL | Least Concern |
|  | *Dipterocarpus borneensis* Slooten | 80 | 6 | DIP.BOR | Near Threatened |
|  | *Dipterocarpus lowii* Hook.f. | 8 | 0 | DIP.LOW | Near Threatened |
|  | *Dipterocarpus palembanicus* Slooten | 2 | 0 | DIP.PAL | Vulnerable |
|  | *Dryobalanops rappa* Becc.* | 19 | 0 | DRY.RAP | Endangered |
|  | *Hopea micrantha* Hook.f.* | 0 | 2 | HOP.MIC | Critically Endangered |
|  | *Hopea pentanervia* Symington ex G.H.S.Wood* | 21 | 5 | HOP.PEN | Vulnerable |
|  | *Hopea* sp.1 | 0 | 2 | HOP.SP.1 | Not Assessed |
|  | *Hopea vaccinifolia* Ridl. ex P.S.Ashton* | 33 | 0 | HOP.VAC | Endangered |
|  | *Shorea havilandii* Brandis* | 29 | 0 | SHO.HAV | Least Concern |
|  | *Shorea longiflora* (Brandis) Symington* | 1 | 3 | SHO.LON | Vulnerable |
|  | *Shorea maxwelliana* King | 3 | 0 | SHO.MAX | Endangered |
|  | *Shorea multiflora* (Burck) Symington | 60 | 0 | SHO.MUL | Least Concern |
|  | *Shorea pachyphylla* Ridl. Ex Symington* | 5 | 0 | SHO.PAC | Endangered |
|  | *Shorea parvifolia* Dyer | 6 | 0 | SHO.PAR | Least Concern |
|  | *Shorea teysmanniana* Dyer ex Brandis | 0 | 2 | SHO.TEY | Endangered |
|  | *Shorea xanthophylla* Symington* | 0 | 5 | SHO.XAN | Near Threatened |
|  | *Vatica brunigii* P.S.Ashton | 1 | 0 | VAT.BRU | Endangered |
|  | *Vatica coriaceae* P.S.Ashton* | 2 | 0 | VAT.COR | Near Threatened |
|  | *Vatica mangachapoi* Blanco | 18 | 0 | VAT.MAN | Vulnerable |
|  | *Vatica micrantha* Slooten | 4 | 0 | VAT.MIC | Least Concern |
|  | *Vatica oblongifolia* Hook.f.* | 2 | 0 | VAT.OBL | Least Concern |
|  | *Vatica parvifolia* P.S.Ashton* | 1 | 4 | VAT.PAR | Vulnerable |
| Ebenaceae | *Diospyros borneensis* Hiern | 1 | 0 | DIO.BOR | Least Concern |
|  | *Diospyros evena* Bakh. | 4 | 3 | DIO.EVE | Not Assessed |
|  | *Diospyros ferruginescens* Bakh.* | 0 | 1 | DIO.FER | Not Assessed |
|  | *Diospyros styraciformis* King & Gamble | 2 | 1 | DIO.STY | Not Assessed |
|  | *Diospyros venosa* Wall. ex A.DC. var. *venosa* | 8 | 16 | DIO.VEN | Least Concern |
|  | *Diospyros* sp.1 | 3 | 2 | DIO.SP.1 | Not Assessed |
| Elaeocarpaceae | *Elaeocarpus griffithii* (Wight) A.Gray | 6 | 1 | ELA.GRI | Not Assessed |
|  | *Elaeocarpus petiolatus* (Jack) Wall | 1 | 0 | ELA.PET | Not Assessed |
| Euphorbiaceae | *Agrostistachys longifolia* (Wight) Benth. & Hook.f. | 2 | 4 | AGR.LON | Not Assessed |
|  | *Blumeodendron tokbrai* (Blume) Kurz | 3 | 2 | BLU.TOK | Least Concern |
|  | *Blumeodendron* sp.1 | 1 | 2 | BLU.SP.1 | Not Assessed |
|  | *Cephalomappa beccariana* Baill* | 3 | 0 | CEP.BEC | Not Assessed |
|  | *Hancea griffithiana* (Müll.Arg.) S.E.C.Sierra, Kulju & Welzen | 1 | 0 | HAN.GRI | Not Assessed |
|  | *Hancea penangensis* (Müll.Arg.) S.E.C.Sierra, Kulju & Welzen | 14 | 14 | HAN.PEN | Least Concern |
|  | *Macaranga beccariana* Merr. | 1 | 0 | MAC.BEC | Least Concern |
|  | *Macaranga conifera* (Rchb.f. & Zoll.) Müll.Arg. | 1 | 0 | MAC.CON | Not Assessed |
|  | *Macaranga gigantea* (Rchb.f. & Zoll.) Müll.Arg. | 9 | 0 | MAC.GIG | Not Assessed |
|  | *Macaranga hosei* King ex Hook.f. | 1 | 0 | MAC.HOS | Not Assessed |
|  | *Macaranga puncticulata* Gage | 10 | 1 | MAC.PUN | Not Assessed |
|  | *Macaranga recurvata* Gage | 5 | 0 | MAC.REC | Least Concern |
|  | *Macaranga trachyphylla* Airy Shaw | 9 | 0 | MAC.TRA | Not Assessed |
|  | *Mallotus* sp.1 | 1 | 0 | MAL.SP.1 | Not Assessed |
|  | *Pimelodendron griffithianum* (Müll.Arg.) Benth. ex Hook.f. | 0 | 2 | PIM.GRI | Not Assessed |
|  | sp. 1 | 1 | 1 | EUP.SP1. | Not Assessed |
| Fabaceae | *Acacia mangium* Wild. | 1 | 0 | ACA.MAN | Least Concern |
|  | *Copaifera palustris* (Symington) de Wit* | 45 | 1 | COP.PAL | Endangered |
|  | *Dialium patens* Backer | 1 | 2 | DIA.PAT | Not Assessed |
|  | *Koompassia malaccensis* Maingay | 5 | 9 | KOO.MAL | Not Assessed |
|  | *Sindora leiocarpa* Backer ex K.Heyne & de Wit | 19 | 16 | SIN.LEI | Least Concern |
| Fagaceae | *Castanopsis foxworthyi* Schottky | 3 | 1 | CAS.FOX | Not Assessed |
|  | *Lithocarpus andersonii* Soepadmo* | 3 | 1 | LIT.AND | Not Assessed |
|  | *Lithocarpus coopertus* (Blanco) Rehder | 12 | 0 | LIT.COO | Least Concern |
|  | *Lithocarpus dasystachyus* (Miq.) Rehder* | 4 | 0 | LIT.DAS | Not Assessed |
|  | *Lithocarpus nieuwenhuisii* (Seemen) A.Camus | 3 | 1 | LIT.NIE | Not Assessed |
|  | *Lithocarpus pusillus* Soepadmo | 1 | 0 | LIT.PUS | Not Assessed |
|  | *Lithocarpus* sp.1 | 8 | 1 | LITH.SP.1 | Not Assessed |
| Hypericaceae | *Cratoxylum glaucum* Korth. | 2 | 0 | CRA.GLA | Not Assessed |
| Lauraceae | *Actinodaphne borneensis* Meisn. | 11 | 15 | ACT.BOR | Least Concern |
|  | *Actinodaphne pruinose* Nees | 0 | 1 | ACT.PRU | Least Concern |
|  | *Actinodaphne* sp.1 | 1 | 0 | ACT.SP.1 | Not Assessed |
|  | *Alseodapne insignis* Gamble | 6 | 1 | ALS.INS | Least Concern |
|  | *Alseodapne* sp.1 | 1 | 0 | ALS.SP.1 | Not Assessed |
|  | *Cinnamomum politum* Miq.* | 0 | 5 | CIN.POL | Least Concern |
|  | *Litsea accedens* (Blume) Boerl. | 1 | 0 | LIT.ACC | Least Concern |
|  | *Litsea cf. ficoidea* Kosterm. | 1 | 0 | LIT.FIC | Vulnerable |
|  | *Litsea elliptica* Blume | 3 | 0 | LIT.ELL | Least Concern |
|  | sp.1 | 0 | 1 | LAU.SP.1 | Not Assessed |
| Lecytidaceae | *Barringtonia reticulata* (Blume) Miq. | 5 | 0 | BAR.RET | Least Concern |
| Magnoliaceae | *Magnolia bintuluensis* (A.Agostini) Noot. | 4 | 0 | MAG.BIN | Data Deficient |
| Malvaceae | *Durio kutejensis* (Hassk.) Becc.* | 2 | 0 | DUR.KUT | Vulnerable |
|  | *Heritiera albiflora* (Ridl.) Kosterm.* | 8 | 13 | HER.ALB | Not Assessed |
|  | *Microcos subcordifolia* R.C.K.Chung* | 2 | 0 | MIC.SUB | Not Assessed |
|  | *Microcos* sp.1 | 1 | 0 | MIC.SP.1 | Not Assessed |
|  | *Pentace rigida* Kosterm.* | 3 | 0 | PEN.RIG | Not Assessed |
| Melastomataceae | *Memecylon borneense* Merr.* | 12 | 2 | MEM.BOR | Not Assessed |
|  | *Memecylon* sp.1 | 1 | 0 | MEM.SP.1 | Not Assessed |
| Meliaceae | *Aglaia glabrata* Teijsm. & Binn. | 7 | 49 | AGL.GLA | Near Threatened |
|  | *Dysoxylum* sp.1 | 1 | 0 | DYS.SP.1 | Not Assessed |
| Moraceae | *Artocarpus odoratissimus* Blanco* | 1 | 0 | ART.ODO | Near Threatened |
| Myristicaceae | *Gymnacranthera farquhariana* (Wall. ex Hook.f. & Thomson) Warb. | 1 | 0 | GYM.FAR | Not Assessed |
|  | *Horsfieldia carnosa* Warb.* | 10 | 3 | HOR.CAR | Near Threatened |
|  | *Horsfieldia crassifolia* (Hook.f. & Thomson) Warb. | 10 | 2 | HOR.CRA | Near Threatened |
|  | *Horsfieldia oligocarpa* Warb. | 16 | 4 | HOR.OLI | Near Threatened |
|  | *Horsfieldia* sp.1 | 6 | 0 | HOR.SP.1 | Not Assessed |
|  | *Knema kunstleri* (king) Warb. | 15 | 1 | KNE.KUN | Least Concern |
|  | *Myristica lowiana* King | 19 | 1 | MYR.LOW | Near Threatened |
|  | *Myristica smythiesii* J.Sinclair | 0 | 1 | MYR.SMY | Not Assessed |
|  | *Myristica* sp.1 | 1 | 0 | TIC.SP.1 | Not Assessed |
| Myrtaceae | *Syzygium acuminatissimum* (Blume) DC. | 0 | 4 | SYZ.ACU | Least Concern |
|  | *Syzygium bankense* (Hassk.) Merr. & L.M.Perry | 7 | 194 | SYZ.BAN | Not Assessed |
|  | *Syzygium beccarii* (Ridl.) Merr. & L.M.Perry | 5 | 2 | SYZ.BEC | Not Assessed |
|  | *Syzygium caryophylliflorum* (Ridl.) Merr. & L.M.Perry* | 2 |  | SYZ.CAR | Vulnerable |
|  | *Syzygium castaneum* (Merr.) Merr. & L.M.Perry | 1 | 1 | SYZ.CAS | Not Assessed |
|  | *Syzygium caudatilimbum* (Merr.) Merr. & L.M.Perry* | 16 | 15 | SYZ.CAU | Not Assessed |
|  | *Syzygium cf. caudatum* (Merr.) Airy Shaw | 1 |  | SYZ.DAT | Not Assessed |
|  | *Syzygium cf. paradoxum* (Merr.) Masam.* | 3 | 1 | SYZ.PAR | Not Assessed |
|  | *Syzygium claviflorum* (Roxb.) Wall. ex Steud. | 2 | 1 | SYZ.CLA | Least Concern |
|  | *Syzygium incarnatum* (Elmer) Merr. & L.M.Perry | 1 |  | SYZ.INC | Not Assessed |
|  | *Syzygium megalophyllum* Merr. & L.M.Perry | 0 | 6 | SYZ.MEG | Not Assessed |
|  | *Syzygium muelleri* (Miq.) Miq. | 14 | 38 | SYZ.MUE | Not Assessed |
|  | *Syzygium tawahense* (Korth.) Merr. & L.M.Perry* | 1 | 5 | SYZ.TAW | Least Concern |
|  | *Syzygium urceolatum subsp. kuchingense* (Merr.) P.S.Ashton* | 2 | 5 | SYZ.KUC | Not Assessed |
|  | *Syzygium urceolatum subsp. palembanicum* (Merr.) P.S.Ashton | 0 | 5 | SYZ.KUC | Not Assessed |
|  | *Syzygium villamillii* (Merr.) Merr. & L.M.Perry* | 0 | 1 | SYZ.PAL | Not Assessed |
|  | *Syzygium* sp.1 | 14 | 8 | SYZ.SP.1 | Not Assessed |
|  | *Syzygium* sp.2 | 0 | 1 | SYZ.SP2 | Not Assessed |
|  | *Tristaniopsis beccarii* (Ridl.) Peter G.Wilson & J.T.Waterh.* | 1 | 0 | TRI.BEC | Not Assessed |
|  | *Tristaniopsis merguensis* (Griff.) Peter G.Wilson & J.T.Waterh. | 2 | 0 | TRI.MER | Not Assessed |
|  | *Whiteodendron moultonianum* (W.W.Sm.) Steenis* | 20 | 0 | WHI.MOU | Not Assessed |
|  | sp. 1 | 1 | 0 | MYR.SP.1 | Not Assessed |
| Ochnaceae | *Brackenridgea palustris* Bartell. | 1 | 1 | BRA.PAL | Near Threatened |
| Olacaceae | *Anacolosa frutescens* (Blume) Blume | 4 | 0 | ANA.FRU | Least Concern |
| Oleaceae | *Chionanthus havilandii* Kiew* | 3 | 0 | CHI.HAV | Not Assessed |
| Pandaceae | *Galearia maingayi* Hook.f. | 2 | 0 | GAL.MAI | Not Assessed |
| Pentaphyllacaceae | *Ternstroemia aneura* Miq. | 23 | 0 | TER.ANE | Not Assessed |
|  | *Adinandra clemensiae* Kobuski* | 3 | 0 | ADI.CLE | Not Assessed |
|  | *Adinandra* sp.1 | 2 | 0 | ADI.SP.1 | Not Assessed |
|  | sp.1 | 0 | 2 | PEN.SP.1 | Not Assessed |
| Phyllanthaceae | *Antidesma coriaceum* Tul. | 6 | 6 | ANT.COR | Not Assessed |
|  | *Aporosa lucida* (Miq.) Airy Shaw | 2 | 0 | APO.LUC | Not Assessed |
|  | *Aporosa* sp.1 | 1 | 0 | APO.SP.1 | Not Assessed |
|  | *Baccaurea bracteata* Müll.Arg. | 7 | 0 | BAC.BRA | Not Assessed |
|  | *Baccaurea* sp.1 | 1 | 0 | BAC.SP.1 | Not Assessed |
|  | *Baccaurea sumatrana* (Miq.) Müll.Arg. | 8 | 0 | BAC.SUM | Not Assessed |
|  | *Cleistanthus baramicus* Jabl.* | 7 | 0 | CLE.BAR | Not Assessed |
|  | *Cleistanthus gracilis* Hook.f. | 28 | 9 | CLE.GRA | Not Assessed |
|  | *Glochidion cf. rubrum* Blume | 2 | 0 | GLO.RUB | Least Concern |
|  | *Glochidion kerangae* Airy Shaw* | 4 | 0 | GLO.KER | Not Assessed |
| Picrodendaceae | *Austrobuxus nitidus* Miq. | 2 | 0 | AUS.NIT | Not Assessed |
| Polygalaceae | *Xanthophyllum affine* Korth. ex Miq. | 4 | 0 | XAN.AFF | Not Assessed |
|  | *Xanthophyllum ferrugineum* Meijden* | 1 | 0 | XAN.FER | Not Assessed |
|  | *Xanthophyllum stipitatum* A.W.Benn. | 3 | 2 | XAN.STI | Not Assessed |
| Primulaceae | *Ardisia copelandii* Mez | 0 | 1 | ARD.COP | Not Assessed |
|  | *Rapanea* sp.1 | 1 | 0 | RAP.SP1 | Not Assessed |
| Rhizophoraceae | *Carallia borneensis* Oliv. | 0 | 1 | CAR.BOR | Not Assessed |
|  | *Pellacalyx lobbii* (Hook.f.) A.Schimp. | 2 | 0 | PEL.LOB | Not Assessed |
| Rosaceae | *Prunus arborea* (Blume) Kalkman | 8 | 0 | PRU.ARB | Least Concern |
| Rubiaceae | *Psydrax* sp.1 | 24 | 7 | PSY.SP.1 | Not Assessed |
| Rubiaceae | *Ridsdalea grandis* (Korth.) J.T.Pereira | 0 | 3 | RID.GRA | Not Assessed |
|  | *Ridsdalea* sp.1 | 2 | 0 | RID.SP.1 | Not Assessed |
| Rutaceae | *Tetractomia tetrandra* (Roxb.) Merr. | 0 | 2 | TET.TET | Least Concern |
| Salicaceae | *Homalium caryophyllaceum* (Zoll. & Moritzi) Benth. | 0 | 2 | HOM.CAR | Not Assessed |
|  | *Homalium moultonii* Merr.* | 0 | 9 | HOM.MOU | Not Assessed |
| Sapindaceae | *Nephelium lappaceum* L. | 0 | 62 | NEP.LAP | Least Concern |
|  | *Nephelium maingayi* Hiern. | 9 | 14 | NEP.MAI | Least Concern |
|  | *Xerospermum laevigatum* Radlk. | 13 | 24 | XER.LAE | Not Assessed |
| Sapotaceae | *Isonandra lanceolata* Wight | 2 | 14 | ISO.LAN | Least Concern |
|  | *Madhuca curtisii* (King & Gamble) Ridl. | 0 | 2 | MAD.CUR | Vulnerable |
|  | *Madhuca pallida* (Burck) Baehni | 1 | 0 | MAD.PAL | Near Threatened |
|  | *Palaquium gutta* (Hook.) Baill. | 1 | 3 | PAL.GUT | Near Threatened |
|  | *Palaquium pseudocuneatum* H.J.Lam* | 1 | 0 | PAL.PSE | Least Concern |
|  | *Palaquium ridleyi* King & Gamble | 8 | 2 | PAL.RID | Least Concern |
|  | *Palaquium* sp.1 | 1 | 0 | PAL.SP.1 | Not Assessed |
|  | *Payena microphylla* (de Vriese) Burck* | 1 | 1 | PAY.MIC | Least Concern |
|  | *Payena obscura* Burck | 1 | 0 | PAY.OBS | Vulnerable |
|  | *Pouteria malaccensis* (C.B.Clarke) Baehni | 6 | 0 | POU.MAL | Near Threatened |
| Simaroubaceae | *Eurycoma longifolia* Jack | 4 | 0 | EUR.LON | Not Assessed |
| Stemonuraceae | *Stemonurus umbellatus* Becc. | 20 | 8 | STE.UMB | Not Assessed |
| Sterculiaceae | *Sterculia rhynchophylla* K.Schum* | 0 | 3 | STE.RHY | Not Assessed |
| Tetrameristaceae | *Tetramerista glabra* Miq. | 1 | 0 | TET.GLA | Vulnerable |
| Thymelaceae | *Gonystylus affinis* Radlk. | 2 | 0 | GON.AFF | Vulnerable |
|  | *Gonystylus calophylloides* Airy Shaw* | 1 | 0 | GON.CAL | Near Threatened |
| Trigoniaceae | *Trigoniastrum hypoleucum* Miq. | 1 | 0 | TRI.HYP | Not Assessed |
| Indet | Species 1 | 1 | 0 | SPP1 | Not Assessed |
| Indet | Species 2 | 1 | 0 | SPP2 | Not Assessed |
| Indet | Species 3 | 0 | 1 | SPP3 | Not Assessed |
