# Supplementary material for: Contrasting environmental drivers of tree community variation within heath forests in Brunei Darussalam, Borneo
Source: Biodivers Data J. 2024 Dec 13;12:e127919. doi: 10.3897/BDJ.12.e127919 (PMC11662205; doi:10.3897/BDJ.12.e127919)
Supplement: Supplementary material 4 — Venn diagram [file bdj-12-e127919-s004.docx]

*
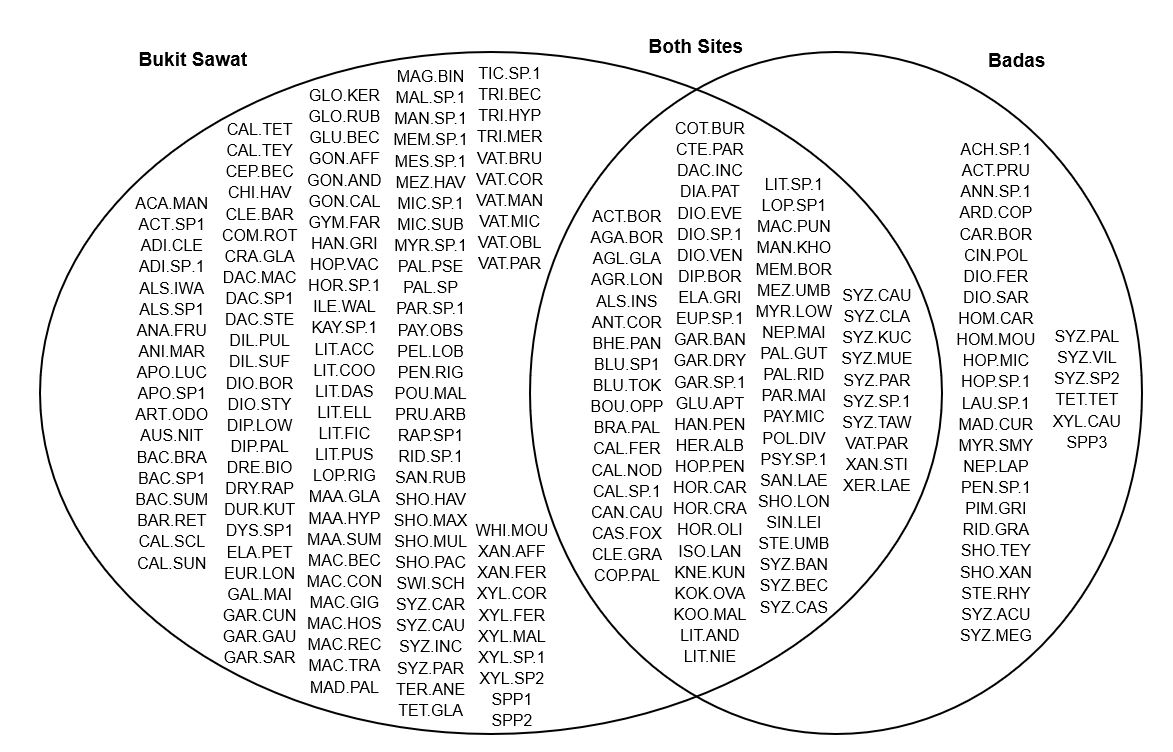
*

Figure S1. Venn diagram showing the species codes for trees with DBH ≥ 5 cm recorded in Bukit Sawat and Badas heath forests arranged alphabetically. All species codes, the full checklist and stem abundance of trees with DBH ≥ 5 cm from Bukit Sawat and Badas are listed in Table S4.
