# Supplementary material for: Contrasting environmental drivers of tree community variation within heath forests in Brunei Darussalam, Borneo
Source: Biodivers Data J. 2024 Dec 13;12:e127919. doi: 10.3897/BDJ.12.e127919 (PMC11662205; doi:10.3897/BDJ.12.e127919)
Supplement: Supplementary material 5 — LME results for species richness [file bdj-12-e127919-s005.docx]

Table S4. Results of ANOVA from linear mixed effects model analysis of species richness and diversity indices (Shannon’s index, Evenness and Inverse Simpson’s index) showing the effects of site. Significant P-values are highlighted in bold.

| Effects | Shannon’s index | | | Evenness | | | Inverse Simpson’s index | | |
| --- | --- | --- | --- | --- | --- | --- | --- | --- | --- |
|  | dF | F | p-value | dF | F | p-value | dF | F | p-value |
| Site | 1 | 95.55 | **< 0.001** | 1 | 57.70 | **< 0.0001** | 1 | 100.10 | **< 0.001** |

| Effects | Species richness | | |
| --- | --- | --- | --- |
|  | dF | F | p-value |
| Site | 1 | 92.55 | **< 0.001** |
