# Supplementary material for: Contrasting environmental drivers of tree community variation within heath forests in Brunei Darussalam, Borneo
Source: Biodivers Data J. 2024 Dec 13;12:e127919. doi: 10.3897/BDJ.12.e127919 (PMC11662205; doi:10.3897/BDJ.12.e127919)
Supplement: Supplementary material 6 — Fits of four environmental properties and eight soil properties on to NMDS ordinations [file bdj-12-e127919-s006.docx]

Table S5. Fits of four environmental properties (humidity, canopy openness, litter depth, elevation, slope and aspect) and eight soil properties (soil pH, soil GWC, total C, N, P, Ca, Mg and K concentrations) onto NMDS ordinations of tree species (based on species abundance) across 48 plots from heath forests at Bukit Sawat (n = 24 plots) and Badas (n = 24 plots). r^2^ values indicate the correlation coefficient denoting the strength of the correlation with p-values assessed using 1000 permutations. Significant p-values (α = 0.05 level; * P < 0.05; ** P < 0.01; *** P < 0.001) are highlighted in bold.

| Environmental properties | Code | Species abundance | |
| --- | --- | --- | --- |
|  |  | r^2^ | P-value |
| Relative humidity | Humidity | 0.1531 | **0.015*** |
| Canopy openness | CO | 0.5612 | **0.001***** |
| Litter depth | LD | 0.0804 | 0.175 |
| Elevation | Elevation | 0.0990 | 0.096 |
| Slope | Slope | 0.5717 | **0.001***** |
| Aspect | Aspect | 0.1100 | 0.089 |
| Soil properties | Code | Species abundance | |
|  |  | r^2^ | P-value |
| Soil pH | pH | 0.1827 | **0.009**** |
| Soil GWC | GWC | 0.4098 | **0.001***** |
| Total C | Total.C | 0.1326 | **0.011*** |
| Total N | Total.N | 0.1253 | **0.019*** |
| Total P | Total.P | 0.1193 | **0.040*** |
| Total Ca | Total.Ca | 0.1365 | **0.025*** |
| Total Mg | Total.Mg | 0.0730 | 0.172 |
| Total K | Total.K | 0.2434 | **0.003**** |
