# Supplementary material for: Contrasting environmental drivers of tree community variation within heath forests in Brunei Darussalam, Borneo
Source: Biodivers Data J. 2024 Dec 13;12:e127919. doi: 10.3897/BDJ.12.e127919 (PMC11662205; doi:10.3897/BDJ.12.e127919)
Supplement: Supplementary material 7 — Pairwise PERMANOVA result test results [file bdj-12-e127919-s007.docx]

Table S6. Pairwise PERMANOVA result test results, based on abundance data, to show pairwise differences in tree community composition between locations: Bukit Sawat (BSAWAT) and Badas FR (BADAS). The PERMANOVA for abundance data was highly significance at p < 0.001 with R^2^ of 0.30.

| Pairwise comparison | *F* | *R*^2^ | p |
| --- | --- | --- | --- |
| BSAWAT vs. BADAS | 21.84 | 0.304 | **0.001**** |
